# Identification of potential biomarkers from microarray experiments using multiple criteria optimization

**DOI:** 10.1002/cam4.69

**Published:** 2013-02-27

**Authors:** Matilde L Sánchez-Peña, Clara E Isaza, Jaileene Pérez-Morales, Cristina Rodríguez-Padilla, José M Castro, Mauricio Cabrera-Ríos

**Affiliations:** 1 Bio IE Lab, Industrial Engineering Department, University of Puerto Rico at MayagüezMayagüez, Puerto Rico; 2 Immunology and Virology Laboratory, Universidad Autónoma de Nuevo LeónMonterrey, México; 3 Integrated Systems Engineering, The Ohio State UniversityColumbus, Ohio

**Keywords:** Cancer biomarkers, cervical cancer, data envelopment analysis, microarray data analysis, multiple criteria optimization

## Abstract

Microarray experiments are capable of determining the relative expression of tens of thousands of genes simultaneously, thus resulting in very large databases. The analysis of these databases and the extraction of biologically relevant knowledge from them are challenging tasks. The identification of potential cancer biomarker genes is one of the most important aims for microarray analysis and, as such, has been widely targeted in the literature. However, identifying a set of these genes consistently across different experiments, researches, microarray platforms, or cancer types is still an elusive endeavor. Besides the inherent difficulty of the large and nonconstant variability in these experiments and the incommensurability between different microarray technologies, there is the issue of the users having to adjust a series of parameters that significantly affect the outcome of the analyses and that do not have a biological or medical meaning. In this study, the identification of potential cancer biomarkers from microarray data is casted as a multiple criteria optimization (MCO) problem. The efficient solutions to this problem, found here through data envelopment analysis (DEA), are associated to genes that are proposed as potential cancer biomarkers. The method does not require any parameter adjustment by the user, and thus fosters repeatability. The approach also allows the analysis of different microarray experiments, microarray platforms, and cancer types simultaneously. The results include the analysis of three publicly available microarray databases related to cervix cancer. This study points to the feasibility of modeling the selection of potential cancer biomarkers from microarray data as an MCO problem and solve it using DEA. Using MCO entails a new optic to the identification of potential cancer biomarkers as it does not require the definition of a threshold value to establish significance for a particular gene and the selection of a normalization procedure to compare different experiments is no longer necessary.

## Introduction

Microarrays are frequently used to simultaneously analyze the expression level of tens of thousands of genes. Analysis of microarray data has become a useful tool for the study of different illnesses including all types of cancer 1–3. Microarray analyses are carried out, essentially, with the objective to detect variation patterns of genetic expression. In cancer research, these patterns can be used for various purposes such as eliciting a diagnosis or prognosis, characterizing a particular illness stage, or detecting and proposing the role of specific genes in the development of cancer. In this last classification, lies the detection of cancer biomarkers. Because biomarker genes detected using only microarray data are not experimentally validated yet, at that point they are deemed potential biomarkers.

Microarray experiments generate large amounts of information whose analysis and interpretation are nontrivial 4. Traditional statistical approaches are challenged by large variances, incommensurability, nonnormality, and the small number or replicates frequently present in these experiments. These challenges hamper finding consistent analysis results 5, thereby leading to a large number of potential biomarkers to be investigated, the research of which could prove lengthy and very expensive.

An example that illustrates the difficulties of obtaining cancer biomarkers consistently is the 70-gene signature for identification of patients with a high probability for breast cancer relapse after its eradication. The original results are reported previously 6. A 76-gene signature is reported in Wang et al. 7 with the same purpose; however, there are only three genes that intersect with the original signature. This issue has been also reported for the specific case of breast cancer by Ein-Dor et al. 8.

It is also notorious that truly integrated work across disciplines is not frequent in most microarray analysis works. Biology and Medicine experts are usually left with the burden of using coded analysis tools with a series of parameters – of statistical, computational, or mathematical nature – that significantly affect the outcome of the software packages 4. This leads to issues in results' reproducibility and comparability between studies.

These challenges motivate the search for microarray analysis techniques from which consistent results can be achieved across several experiments and researches, particularly for the identification of potential cancer biomarkers. In this study, a multiple criteria optimization (MCO) approach is proposed for the identification of potential cancer biomarkers from microarray data. An MCO problem aims to find the best compromises between two or more conflicting criteria 9. The best compromises are located in the so-called Pareto-efficient frontier. It is proposed that the genes in the efficient frontier of the MCO problem, built with performance measures relating to the significant change in gene expression, are potential cancer biomarkers.

The potential of an MCO analysis for the identification of relevant genes has been recognized before 10 through the use of ranking methods. Here, the proposed MCO problem is solved through the use of data envelopment analysis (DEA) 11. DEA has been used to find the convex efficient frontier of MCO problems 12. DEA is a very computationally convenient technique that is capable to deal with multiple and incommensurable performance measures. A clear applicability to meta-analysis follows from these characteristics. Using MCO provides a new optic to the identification of potential cancer biomarkers as it does not require the definition of a threshold value to establish significance for a particular gene and the selection of a normalization procedure to compare different experiments is no longer necessary.

The proposed method is tested here through its initial application to a microarray database related to cervix cancer 13 and the results are successfully validated through the information available in the literature for the selected genes. Furthermore, two additional studies involving two independent experiments using the same microarray 14,15 platform further corroborate the performance of the proposed method. Finally, the novelty of this approach is contrasted with the use of a single criterion – or performance measure – to find potential biomarkers.

## Methods

### Potential biomarkers through MCO

**Figure 1:**
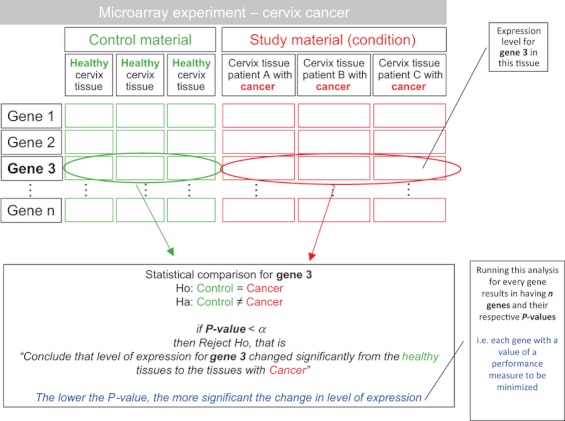
Schematic example of how to obtain a *P*-value. This is a schematic example of how to obtain one *P*-value for a particular gene in a microarray experiment with *l* = 3 healthy tissues as controls and *m* = 3 tissues with cancer. If statistical comparison is carried out for each gene, then at the end one has *n* genes each one with an associated *P*-value.

In microarray experiments, it is critical to be able to quantify changes in genetic expression. A series of measurements have been proposed in the literature that include variations of pure magnitude of relative change of expression versus a control 16 as well as *P*-values obtained from various statistical tests 17. A *P-*value, in statistical comparison procedures, can be understood as the probability associated with finding – by pure chance – a difference in the populations being compared that is at least as large as the observed difference of the samples involved. Lower *P*-values indicate larger differences and therefore show stronger evidence favoring statistical significance. Due to their interpretation capabilities, *P*-values have been a favored performance measure in microarray experiments in recent years. Obtaining a *P*-value for a particular gene is illustrated in Figure 1.

**Figure 2:**
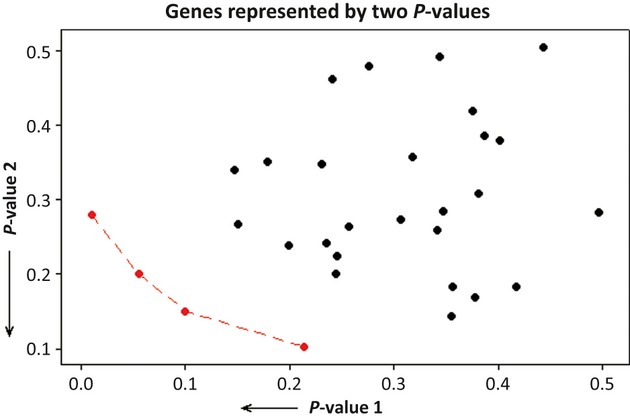
Pareto-efficient frontier. The existence of conflict causes that different genes be attractive when lying in the southwest envelope of the gene set. In general, in multiple criteria optimization (MCO), that envelope is called a Pareto-efficient frontier and it is conformed by Pareto-efficient solutions.

A *P*-value, when obtained for a particular gene in a microarray experiment, can be thought of as a criterion to be minimized since the smaller the *P*-value the more important the change in expression of the gene under consideration. Now, if more than one *P*-value is available for a particular gene, then the task at hand is one of multiple criteria minimization. An illustrative example with a series of genes is shown in Figure 2. In this figure, each gene is represented by a pair of *P*-values. Because low *P*-values are attractive, the ideal gene would be found in the southwest corner of the graph. When no single gene is best in all criteria under consideration, a conflict exists.

The key idea in this study is that the potential biomarker genes can be identified as efficient solutions of the MCO problem that results from representing each gene under analysis through a series of associated *P*-values. In order to develop the idea, two issues must be addressed (i) how can one obtain several *P*-values for one gene? and (ii) which method can be used to solve the MCO problem.

### Obtaining multiple *P*-values for a particular gene

Consider the results of a microarray experiment laid out on a table where the first column contains the names of the *n* genes under study; the columns to the right contain the measurements for *l* healthy tissues followed by *m* cancer tissues. Thus, for each gene, there are *l* replicated measurements of relative expression for state 1 (healthy) and *m* replicates for state 2 (cancer).

A statistical comparison procedure can be used to obtain a *P*-value when contrasting parameters from the two states – cancer and healthy – for a particular gene. A common interest is to compare the population centers, which are estimated either through sample means or sample medians. For MCO purposes, however, more than one *P*-value per gene is necessary. Two cases can be distinguished here: (c1) having a single microarray experiment to study one type of cancer and (c2) having several microarray experiments to study one type of cancer. In c1, if a leave-one-out strategy is applied to the tissues pertaining to one state, then it is possible to obtain several *P*-values. In c2, an additional *P*-value can be obtained for the genes that are common to both experiments. This study focuses on c1 to introduce the proposed analysis strategy, leaving c2 for future publication.

For c1, the leave-one-tissue-out strategy implies extracting a particular tissue associated with one state (“leaving one column out”). By removing a vector (column), a replicate is deleted from the set, thereby forcing a *P*-value that is different from the original one. Thus, two different *P*-values are effectively created. The selection of the tissue to be removed to create a distinct matrix is performed considering the variance of expression on each tissue (stored in each column). Then, a first matrix is built leaving out the tissues (columns) with the highest variance for each state and the second matrix by leaving out the tissues with the lowest variance for each state. Through this strategy, the resulting matrices show extreme cases in terms of data variance. Any other combination of tissues to leave out would have statistical differences lying between these two “extreme” cases.

Thus, two extreme cases span all the possible cases in terms of variance for the leave-one-out cases. This fact can be used to avoid unnecessary computational effort and, by using just two dimensions, it is possible to illustrate the problem graphically.

c1 is important because the vast majority of published microarray experiments are instances of this type, and – as explained previously – it is the subject of study in this manuscript. c2 can be built from several c1 instances, however, it is envisioned that this case becomes an archetype for a study designed to keep the same genes throughout all microarrays experiments involved. c2 will also represent the case where meta-analysis must be addressed and will be approached in a future publication.

### Solving the MCO problem

The decision that must result from the solution of the MCO problem can be stated as “a selection of those genes that show the highest possible expression change in all experimental instances when considered simultaneously.” Due to the large variability encountered in microarray experiments, this is a nontrivial decision that will lead to a set of genes that will have very low *P*-values in certain instances, although not necessarily in all of them, that is, the genes that are Pareto-efficient as illustrated in Figure 2.

DEA is a technique that has been shown capable to identify the efficient solutions located in the convex hull of an MCO problem 11. In its most popular form, DEA finds the Pareto-efficient solutions through the sequential solution of a series of linear optimization models. One of the most popular and effective DEA formulations is the Banker–Charnes–Cooper model (BCC), which is shown next in its two formulations (input oriented and output oriented):

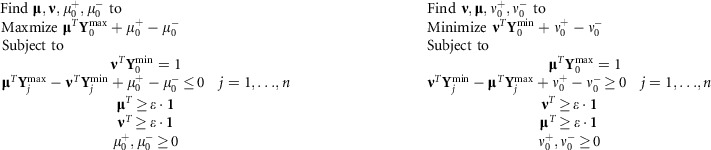

where **μ** and **ν** are vectors containing nonnegative multipliers and 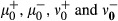 are scalar numbers to be determined optimally, 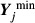 and 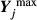 are vectors containing the values of performance measures to be minimized and maximized, respectively, for the *j*th solution. The subindex 0 is used to denote the solution currently under analysis, and *ε* is a small constant usually set to a value of 1 × 10^−6^. The results of solving these two linear optimization problems, for the *n* genes in a set, are a series of hyperplanes that forms a convex envelope around this set, as depicted in Figure 2.

Because of the nature of DEA, the model needs at least one performance measure to be maximized. For the case under consideration, a transformation of at least one set of *P*-values is required. The following transformation is applied to switch from minimization to maximization in a set of *n P*-values:

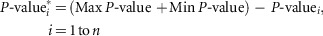
(1)
where the transformation is carried out for the *i*th gene. Maximizing the transformed performance measure is fully equivalent to minimizing the original *P*-value.

DEA has several advantages including (i) computational efficiency owing to its linear optimization structure; (ii) objectivity and consistency of results, which follows from not requiring the adjustment of parameters or assigning weights to the different performance measures; and (iii) capability of analyzing several microarray experiments with incommensurate units. Furthermore, linear optimization is – by far – the most coded type of optimization. Algorithms for linear programing (as this type of optimization is known as) are available in modules from the very common MS Excel package to the mathematically oriented software Matlab 18 and to the very specialized solvers like Lingo 19. There are also DEA solvers like DEA Solver Pro 20 that make adopting the proposed approach even easier. So, in order to use the approach proposed here, all the user needs is a list of genes, with one *P*-value obtained as usual, and a second *P*-value transformed using equation (1), and an optimization solver capable to deal with linear programing to use the DEA formulations outlined above.

One limitation of DEA is that of depending on a series of local linear approximations, as shown in Figure 2. Every time that a hyperplane is superimposed over the set under analysis, there are genes lying in the nonconvex part of the set frontier that escape detection. These genes could be potential biomarkers, however.

**Figure 3:**
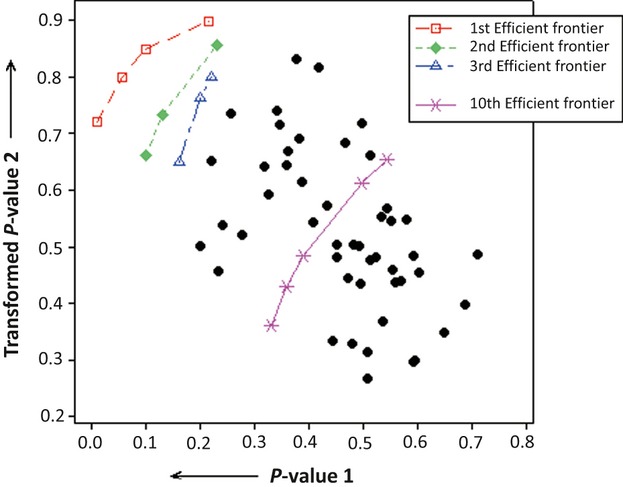
The two performance measures for each gene. This figure schematically shows a case with genes characterized by two performance measures: an untransformed *P*-value and a transformed one with equation (1). Referring to this figure, and following the proposed method, at this point it is recommended to identify the first 10 efficient frontiers. This can be easily done by identifying the genes in the first efficient frontier through data envelopment analysis (DEA), then removing them from the set and continuing with a second DEA iteration. This is repeated until the tenth frontier is identified. A method to determine the number of adequate frontiers to be analyzed is currently under development by our research group.

In order to circumvent this limitation, it is proposed that DEA be applied successively 10 times, each time removing the genes found in a particular iteration from the set for subsequent analyses. This strategy results in 10 frontiers, as seen in Figure 3.

## Results

### Analysis of a single microarray experiment to study one type of cancer

The first results on the application of the proposed method include the analysis of the microarray database used by Wong et al. 13 related to cervix cancer. The database consists of eight healthy tissues and 25 cervix cancer tissues, all of them with expression level readings for 10,692 genes from a cDNA microarray. The Mann–Whitney nonparametric two-sided test for comparison of medians was used to generate two different *P*-values per gene 21, following the leave-one-tissue-out strategy as outlined in the methods section. Both formulations were applied to each gene characterized by a *P*-value as an input and as a transformation of the other *P*-value as an output (equation 1). The first 10 frontiers were identified, and they contained 28 potential biomarkers. Numerically, reducing 10,692 genes to only 28 of them evidences the screening power of the proposed method. Table 1 outlines the genes identified in the analysis. These were then investigated in the literature to assess their cervix cancer biomarking potential as discussed next.

**Table 1:** List of the 28 genes identified in the first 10 frontiers of the proposed multiple criteria optimization (MCO) problem

Frontier	Accession number	Symbol	Name	Expression in cervix cancer (using data from Wong et al. 13)
1	AA488645	*NAB1*	NGFI-A-binding protein 1 (EGR1 binding protein 1)	Underexpressed
2	H22826	*LMO7*	LIM domain 7	Overexpressed
3	AI553969	*KPNA6*	Karyopherin α6 (importin α7)	Overexpressed
3	T71316	*ARF4*	ADP-ribosylation factor 4	Overexpressed
3	AA243749	*DDR2*	Discoidin domain receptor tyrosine kinase 2	Overexpressed
3	AA460827	*PPP1R1A*	Protein phosphatase 1, regulatory (inhibitor) subunit 1A	Underexpressed
4	AA454831		EST: zx79c10.s1	Overexpressed
4	AA913408, AA913864	*RAD52*	DNA damage repair and recombination protein RAD52 pseudogene	Overexpressed
5	AA487237	*UBE3A*	Ubiquitin protein ligase E3A	Underexpressed
5	AA446565	*RBM25*	RNA-binding motif protein 25	Overexpressed
6	H23187	*CA2*	Carbonic anhydrase II	Overexpressed
7	AI221445	*KCNE3*	Potassium voltage-gated channel, Isk-related family, member 3	Overexpressed
7	R36086		EST: yh88d01.s1	Underexpressed
7	AA282537	*LOC729991*	Hypothetical protein LOC729991	Overexpressed
8	N93686	*ALDH3B1*	Aldehyde dehydrogenase 3 family, member B1	Underexpressed
8	R91078	*CYP3A7*	Cytochrome P450, family 3, subfamily A, polypeptide 7	Overexpressed
8	R44822	*PRPSAP1*	Phosphoribosyl pyrophosphate synthetase-associated protein 1	Underexpressed
9	AI334914	*ITGA2B*	Integrin, alpha 2b (platelet glycoprotein IIb of IIb/IIIa complex, antigen CD41)	Overexpressed
9	R93394		Transcribed locus	Overexpressed
9	AA621155	*MSH5*	MutS homolog 5 (*Escherichia coli*)	Underexpressed
9	AA705112	*MOCS1*	Molybdenum cofactor synthesis 1	Overexpressed
9	R52794	*PTPRT*	Protein tyrosine phosphatase, receptor type, T	Underexpressed
10	AA424344	*UROD*	Uroporphyrinogen decarboxylase	Overexpressed
10	H69876	*LOC100132707*	Hypothetical LOC100132707	Underexpressed
10	H55909	*SRSF1*	Serine/arginine-rich splicing factor 1	Underexpressed
10	W74657	*KLF2*	Kruppel-like factor 2 (lung)	Overexpressed
10	AI017398	*ACCN2*	Amiloride-sensitive cation channel 2, neuronal	Overexpressed
10	H99699	*POLR3H*	Polymerase (RNA) III (DNA directed) polypeptide H (22.9 kD)	Overexpressed

The table shows complete list of genes identified in the first 10 efficient frontiers. In the last column, the expression change from the normal state to the cancer state is shown.

In the first efficient frontier there is only one gene: the *NAB1* gene that codes for EGR1-binding protein 1, which has been reported as a potential tumor suppressor in different cancer types including prostate cancer 22, breast cancer 23, esophageal cancer 24, hepatoma 25, and leukemia 26.

The LIM domain 7 (*LMO7*) gene was selected in the second frontier. The protein product of the *LMO7* belongs to the PDZ-LIM family. Regulation problems with these proteins can support the development of cancer 27.

Third frontier holds *DDR2*, *PPP1R1A*, *ARF4*, and *KPNA6*. Changes in expression of *DDR2* have been linked to several human cancers, for example, in non-small cell lung carcinoma (NSCLC) 28 and in nasopharyngeal carcinoma 29. The *PPP1R1A* product is the protein phosphatase 1, regulatory (inhibitor) subunit 1A. In a recent study, the *PPP1R1A* expression in lung, colorectal, and gastric cancer cell lines was different from that of the normal tissues 30, as well as in some cell lines developed from different pediatric tumors 31. The ADP-ribosylation factor 4 (*ARF4*) gene protein product interacts with epidermal growth factor receptor (EGFR) mediating the EGF-dependent cellular activation of phospholipase D2 (*PLD2*) 32. An increased *PLD2* activity has been reported for human cancers including breast, colon, gastric, and kidney 33. The *ARF4* has also been proposed as an antiapoptotic gene in human glioblastoma-derived U373MG cells 34. The product of the *KPNA6* gene has been reported to play an important role in the antioxidant response and in keeping the redox homeostasis of the cell 35. Its downregulation was reported to inhibit HeLa cell proliferation 36.

The fourth frontier holds *RAD52* along with an expressed sequence tag (EST). *RAD52* codes for a protein that is homolog to the *Saccharomyces cerevisiae* Rad52. The overexpression of *RAD52*, along with *RAD51* and *TOP2A*, all three DNA repair genes, has been reported to be predictive of poor relapse-free survival for melanoma 37.

The genes in the fifth frontier are *RBM25* and *UBE3A*. The product of the *RBM25* gene is an RNA-binding protein that acts as a splicing factor and has been shown to act on the alternative splicing of apoptotic factors 38. The product of the *UBE3A* gene is an E3 ubiquitin protein ligase, the E6-associated protein (E6AP). This protein is used by the E6 oncoprotein, from high-risk human papillomavirus (HPV) types, to produce the proteolysis of the tumor suppressor p53 39. The E6AP is also used by E6 to stimulate the telomerase activity, generally present in cancer cell lines 40.

CA II, the gene in the sixth frontier, has been reported to be expressed in the neovessel endothelium and the tumor cell cytoplasm of medulloblastomas and primitive neuroectodermal tumors 41 and has been proposed as a biomarker gene for gastrointestinal stromal tumors 42.

In the seventh frontier *KCNE3*, the uncharacterized conserved protein LOC729991, and the EST yh88d01.s1 were selected. The *KCNE3* gene codes for the potassium voltage-gated channel, Isk-related family, member 3. An increase in the activity of plasma membrane voltage-gated potassium channels promote neuronal cell death by apoptosis 43.

The genes in the eighth frontier are *ALDH3B1*, *CYP3A7*, and *PRPSAP1*. In a recent study, the expression of *ALDH3B1* was found to be tissue dependent, being upregulated in a high percentage of tumors used in the study (lung > breast = ovarian > colon) 44. *CYP3A7* codes for a protein from the cytochrome P450 superfamily of enzymes. Proteins of this family play an important role in carcinogenesis because they metabolically activate precarcinogens and can metabolize anticancer drugs. The product of the *PRPSAP1* gene has been suggested to play a negative regulatory role in 5-phosphoribose 1-diphosphate synthesis and to bind to *PRPS1* and *PRPS2* [Bibr b45], enzymes involved in the synthesis of purine and pyrimidine nucleotides.

The genes in the ninth frontier are *ITGA2B*, *MSH5*, *MOCS1*, and *PTPRT*. The *ITGA2B* gene codes for the integrin alpha chain 2b. Integrins can activate protein kinases involved in the regulation of cell growth, division, survival, differentiation, migration, and apoptosis. The *MSH5* gene codes for a member of the mutS family of proteins. These proteins are involved in promoting ionizing radiation-induced apoptosis 46. A recent study found that the level of mRNA for genes involved in mismatching repair, including *MSH5*, was lower in colorectal cancer samples than in normal tissues 47. The product of the *MOCS1* gene is involved in the molybdenum cofactor biosynthesis. Deficiency in molybdenum cofactor produces deficiency in the sulfite oxidase, xanthine dehydrogenase, and aldehyde oxidase 48. Xanthine oxidoreductase has been associated with various forms of cancers as well as other human diseases (reviewed in 49). The *PTPRT* gene codes for a tyrosine phosphatase protein, receptor type T, and has been suggested that its product has tumor suppression functions 50.

In the 10th frontier, the genes selected by the analysis method used in this study are *UROD*, *LOC100132707*, *SRSF1*, *KLF2*, *ACCN2*, and *POLR3H*. The *UROD* gene has been reported to be overexpressed in biopsies from patients with head and neck cancer 51. *LOC100132707* is a hypothetical gene, the product of which is uncharacterized. The *SRSF1* gene codes for a member of the arginine/serine-rich splicing factor protein family, its product works activating or repressing splicing of pre-mRNA [Bibr b52]. It has been proposed that *KLF2* could have a tumor suppressor activity in the MCF-7 mammary carcinoma cells 53. Also, the expression of *KLF2* has been reported to inhibit Jurkat T leukemia cell growth 54. The *ACCN2* product is an acid-sensing ion channel (ASIC) shown to have higher expression in human glioblastoma multiforme cells as compared with primary human astrocytes 55. The *POLR3H* gene codes for the polymerase (RNA) III (DNA-directed) polypeptide H. RNA polymerase (pol) III synthesizes several products required for protein synthesis, and there have been detected high rates of pol III transcription in several cancers (reviewed in 56).

As it can be seen, the literature marshaled about the genes detected by the proposed method evidences the biological relevance of the analysis output. The following section presents cross-validation studies that support analysis consistency.

### Cross-validation studies of results in cervix cancer

The proposed method is capable to importantly accelerate the detection of potential cancer biomarkers, as shown in the previous study. In the following studies, the objective was to cross-validate the use of the method following (1) a genetic signature approach and (2) a statistical classification procedure.

Two independent cervix cancer databases using the same microarray platform, the Affymetrix U133A (with 22,283 probe set), were identified [14, 15]. Using the proposed method as in the previous study, and considering only the healthy and cancer data, a series of potential biomarkers was selected using solely database 1 14. These genes were then identified in database 2 15 and the change in expression was compared between the datasets. Table 2 shows the overlap between the reference signature behavior from database 1 and the behavior of genes in database 2. The overlap amounts to 28 genes (29 probes with two probes for gene *SMC4*), which is 71.8% of the original signature, evidencing the effectiveness of the method. Table 2 also summarizes evidence found in the literature to support the genes' potential biomarking role in cervix cancer or in other types of cancer.

**Table 2:** List of genes from the cross-validation study

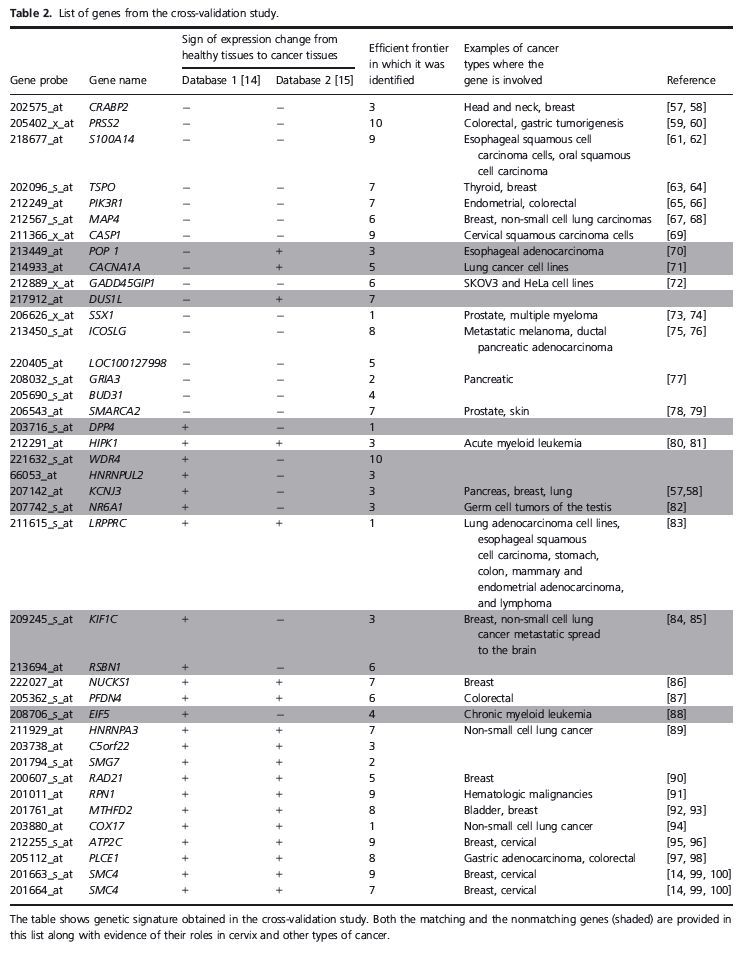

An important fact to emphasize in this study is, also, that of the discrimination power of the tool. The microarray platform used by both databases involved in the validation study contained 22,283 probes set. The fact that a signature of 39 genes was feasible to be built and tested evidences the advantage of using the proposed method.

A second cross-validation study entailed building a linear classifier with the set of genes identified as potential biomarkers in database 1, but applying it to classify the tissues in database 2. The classification rate in the 56 tissues of database 2 (24 healthy tissues and 32 cancer tissues) was 100%. The classifier was built with linear discriminant analysis and the results imply that the selection of potential biomarkers in database 1 achieved perfect linear separability in database 2. This provides solid evidence on the competitiveness of the proposed method.

### Contrast with the single performance measure strategy

The single performance measure strategy is prevalent in the literature for the selection of genes that change their expression significantly between the conditions under comparison. It generally involves defining a threshold to select a number of potential biomarkers based on a single measurable criterion. The definition of such threshold may vary from experimenter to experimenter, however.

In this section, a multiple simultaneous hypothesis testing approach with a Bonferroni correction by Holms 101 was used to contrast a single performance measure strategy with the multiple performance measure strategy proposed here. For each gene in database 1 14, a *P*-value was obtained based upon the Mann–Whitney nonparametric test for difference of medians between two groups. All genes and their associated *P*-values were sorted in increasing order in terms of *P*-value. To decide whether a gene in the (*i*)th place of the ordered sequence shows significantly different relative expression levels with the presence of cancer, the following criterion is evaluated:

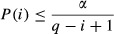
(2)
where *α* is the family-wise error rate and *q* is the number of total hypothesis tests being carried out, which in this instance, corresponds to the number of genes under evaluation.

The choice of the value of *α* is habitually left to the user. With database 1, when *α* < 0.1280, no gene is deemed to change its relative expression significantly. At *α* = 0.1280, a total of 86 genes are deemed to have changed their relative expression significantly. The number of genes in this category goes up to 116 at *α* = 0.1530. The choice of *α* by the user, as it can be seen, greatly affects the number of genes that are considered important.

To make a fair comparison with the proposed multiple criteria method in this study, only the top 39 genes were chosen to build a linear classifier to be applied to database 2 15 as in the previous section. The classification rate was also of 100% in both healthy tissues and cancer tissues. It is important to notice that although both methods achieved 100% classification rate in an independent database, the proposed multiple criteria method did not require for the user to set any parameter.

## Conclusions

The search for potential cancer biomarkers can be greatly enhanced through the use of optimization techniques. In this study, a multiple criteria representation of the gene expression changes identification problem using microarray data is proposed. As a first case, the analysis of a single microarray experiment has been used to extract biologically relevant information in terms of potential biomarkers. The methodology can be extended to find the best compromises between data from different experiments for the same cancer type.

DEA is shown as a promising first approach to characterize the convex-efficient frontier of the MCO problem, and therefore to point toward potential biomarkers in a parameter-free and consistent fashion.

The proposed method, when applied to a publicly available microarray database from cervix cancer, identified genes already reported as relevant for different cancer types or cellular processes related to cancer. When the behavior of a selected gene was contrary to what was expected (*NAB1* [AA488645], *RBM25* [AA446565], *UBE3A* [AA487237], *ALDH3B1* [N93686], *PRPSAP1* [R44822]), the original data were reexamined. For those genes the readings showed great dispersion, from one run to the next, making the signal very noisy, which can explain the odd observed behavior. Genes without previous report of their relevance can be proposed for further *in vitro* validation.

Similarly, in the cross-validation studies, 39 genes were identified as potential cervix cancer biomarkers in a database. Of these genes, there was an overlap of 29 genes with similar behavior in a second database using the same microarray platform. These genes are proposed in this study as potential cervix biomarkers. A second cross-validation study showed that the proposed selection of potential biomarkers achieved perfect linear separability in an independent database, adding evidence in favor of the performance of the proposed approach. Furthermore, the convenience of not requiring the user to set parameters that affect the output of the analysis was demonstrated through a comparison with a commonly used strategy based on a single performance measure.

New methodologies for biological characterization have emerged after microarrays. The issues in handling large amounts of data, analysis reproducibility, and consistency, as well as computational convenience will continue to be challenges. This situates the proposed approach as a promising tool capable to accelerate biological discovery and to facilitate meta-analysis.
